# The progression of the tobacco epidemic in India on the national and regional level, 1998-2016

**DOI:** 10.1186/s12889-021-12261-y

**Published:** 2022-02-15

**Authors:** Rufi Shaikh, Fanny Janssen, Tobias Vogt

**Affiliations:** 1 International Institute for Population Sciences (IIPS), Mumbai, India; 2 Netherlands Interdisciplinary Demographic Institute-KNAW/University of Groningen, Groningen, The Netherlands; 3 Population Research Centre, Faculty of Spatial Science, University of Groningen, Groningen, The Netherlands; 4 Max Planck Institute for Demographic Research, Rostock, Germany; 5 Prasanna School of Public Health, Manipal Academy of Higher Education, Manipal, India

**Keywords:** Tobacco consumption, Epidemic, India, Smoking, Smokeless

## Abstract

**Background:**

Evidence regarding the progression of the tobacco epidemic remains fragmented in low- and middle-income countries. In India, most of the studies that examined tobacco consumption focused on one time point, on the country as a whole, and on men. Despite important gender differences in tobacco consumption, vast economic and cultural differences exist within India. We, therefore, assessed the progression of the tobacco epidemic in India on both the national and the regional level, by gender.

**Methods:**

We use information on current tobacco use among Indians aged 15–49 from three rounds of the National Family Health Survey (NFHS) (1998-99, 2005-06, 2015-16) to estimate the age-standardized sex specific smoking and smokeless tobacco prevalence across India and its states.

**Results:**

Age-standardized tobacco use prevalence in India increased between 1998-1999 and 2005-2006, and declined from 2005-2006 to 2015–2016, simultaneously for men and women. There are substantial spatial differences in the progression of the tobacco epidemic in India. While tobacco use declined in the majority of states, we observe high and increasing use for men in the north-eastern states of Manipur, Mizoram and Nagaland, and for women in the western state of Gujarat and north-eastern state of Manipur. We observed even more states with a recent increasing prevalence in either tobacco smoking or smokeless tobacco. Throughout, prevalence of tobacco use has been higher among men than women for all Indian regions, and remained higher than the national average in the north-eastern states.

**Conclusions:**

Our results suggest that India and the majority of its states experienced a ‘compressed tobacco epidemic’ in which the prevalence of tobacco consumption increased and decreased simultaneously for women and men over a comparatively short period of time. Despite the overall progress India made in reducing tobacco use, further lowering tobacco consumption remains a public health priority, as the prevalence of smoking and/or smokeless tobacco use remains high in a number of states. We therefore conclude that tobacco regulations should be expanded with the aim of reducing the overall health burden associated with tobacco consumption across India.

**Supplementary Information:**

The online version contains supplementary material available at 10.1186/s12889-021-12261-y.

## Background

Tobacco consumption is the single most preventable cause of death and disability across the globe [1, 2]. In 2010, WHO estimated that 4.9 million premature deaths per year are attributable to tobacco use, mostly in the form of smoking [2]. This number rose to 7.1 million in 2016, and is expected to reach eight million by 2030 if the current rate of tobacco consumption is unchanged [3]. The health burden of tobacco consumption is disproportionately high in developing countries. It has been estimated that in these countries, tobacco consumption will kill around 40 million people in total between 2005 and 2030 [4]. Since smoking is the dominant form of tobacco consumption in developed countries, the progression of smoking prevalence and the expected health damage due to tobacco use have been framed within the smoking epidemic model [5, 6]. According to this framework, societies undergo successive stages in which the prevalence of smoking first increases, and then declines. It is, however, striking that evidence on the progression of the smoking epidemic is extensive for developed countries, but remains fragmented for low- and middle-income countries like India [5]. The movement of the smoking epidemic from high-income to low- and middle-income countries may lead to an unprecedented level of premature mortality, posing one of the biggest preventable public health threats to current and future world health [6].

As the country with the second-highest level of tobacco consumption worldwide [7–9], India faces a particularly large health challenge. In 2017, approximately 266.8 million adults in India used tobacco in one form or another [7]; a figure that is more than twice as high as in the European Union [10]. Because of the health risks and health care costs, tobacco use has been framed as an epidemic in itself. Cigarette smoking is less common in India than it is in western countries and most of the tobacco consumed is in the form of bidi smoking [8] or smokeless tobacco [7], making cigarette smoking only one component of overall tobacco consumption in the country [11]. According to a 2008 study by Jha et al. [12], deaths of more than one million adults per year in India can be attributed to different forms of tobacco smoking. The use of smokeless tobacco is also considered a major health risk in India, and has been shown to be associated with an increased risk of death [11, 13–19].

Most existing studies on tobacco use in India have focused on tobacco prevalence at a single point in time, either for the country as a whole or for specific localized settings, and with a focus on men [11, 15, 20–24]. In addition, most of these studies were based on non-representative sample surveys, and did not consider different types of tobacco products consumed [15, 18, 21, 22, 25–27].

Only three previous studies looked into trends of tobacco use over time [28–30], one study focusing on India [28], and two examining different states in India [29, 30]. In a comparative study for South Asian countries, Sinha et al. (2005) estimated national level age-specific trends in smokeless tobacco for India from 1998 to 2010. Mishra et al. (2016) estimated trends in age-standardized cigarette and bidi smoking between 1998 and 2010 across Indian states by age, gender, and education. The study combined smaller north-eastern states and three other major states, and majority of the results focused on men. Goel et al. (2014) estimated trends in combined tobacco smoking prevalence among women in India from 1993 to 2009. Each of these studies used combinations of different cross-sectional surveys over time to estimate trends in tobacco use. These surveys have varying sampling designs and are not representable on a regional level. Therefore these previous estimates may not be as reliable and comparable over time.

Previous studies documented a reduction of tobacco consumption on the national level but comprehensive studies on the regional level that equally focus on men and women are largely missing. India has 29 states and 7 union territories with larger social differences and cultural habits [24, 31, 32] and tobacco use is ingrained as a cultural practice. These differences may result in regional disparities in the tobacco consumption prevalence and a non-synchronous progression in the tobacco pandemic that is not reflected at the national level. A focus on the regional level can also add to discussion whether tobacco control policies have bought reduction in tobacco prevalence across different Indian regions.

Our study therefore aims to fill this gap and contribute to the scientific discussion by estimating the progression of the tobacco epidemic in India by gender and across all Indian states. To provide a comprehensive overview of tobacco use in India and its states, we include both trends in tobacco smoking and in use of smokeless tobacco. By incorporating smokeless tobacco into the classical smoking epidemic model, we do not only account for different forms of tobacco consumption and how they have changed over time, but also for country-specific peculiarities in tobacco consumption patterns. In addition, our study is the first to use a high-quality dataset to apply the tobacco epidemic model to India and its states in order to discern national and subnational patterns in the progression of tobacco prevalence. Though descriptive in nature, the findings of our study have great value as they help in monitoring population dynamics at the national and regional level and highlight regions in need of public health action. The study goes beyond the previous established studies and provides a comprehensive overview of tobacco use in India on a national and regional level, which was not done yet. Various public health initiatives aimed at reducing tobacco use have been implemented in the country since 1975. We discuss whether these public health initiatives have affected female and male tobacco consumption over time, and conclude that tobacco regulations should be expanded with the aim of reducing the overall health burden of tobacco consumption across India.

## Methods

### Design/Data

We use data from second, third, and fourth rounds (1998–2016) of the National Family Health Survey (NFHS) to estimate national- and state-level age-standardized tobacco prevalence for men and women aged 15–49. The NFHS is a nationally representative, cross-sectional household and individual sample survey that represents 99% of the Indian population living in 27 states and two union territories [33–35]. It is the only population survey in the country that provides reliable estimates for various socio-demographic, lifestyle and morbidity indicators, for females aged 15–49 and males aged 15–54. In the NFHS-2, conducted in 1998-99 with a response rate of 97%, 91,196 households were interviewed. In the NFHS-3, undertaken in 2005-06 with a response rate of 98%, 109,041 households were questioned. In the NFHS-4, conducted in 2015-16 with a response rate of 98%, 601,509 household were interviewed.

### Measures

Information on tobacco use of household members was collected through self-administered questionnaires that included the following four questions: 1) “Do you currently chew pan masala or tobacco?” “Do you currently smoke cigarettes or bidis?” “Do you currently smoke or use tobacco in any other form?” “In what other forms do you currently smoke or use tobacco?” These questions were answered by the head of the household in the 1998-99 round, and by each individual respondent in the other two rounds. We categorized individuals as “tobacco users” if the respondent answered “yes” to either one of the first three questions, and thus combined tobacco smoking and smokeless tobacco use. In addition, we distinguished between tobacco smoking (smoking of cigarettes, bidis, pipes/hookah, and other items) and smokeless tobacco use (consumption of products like ghutka, pan masala, snuff, and khaini).

### Analyses

We calculated the prevalence of age-specific tobacco use, tobacco smoking, and smokeless tobacco use for men and women aged 15–49 years for the years 1998-99, 2005-06, and 2015-16 for India and its 27 states and 2 union territories.

For the estimation of representative national and sub-national age-specific tobacco use prevalence, we applied appropriate sampling weights that account for the multistage cluster sampling design of the NFHS. The sampling weights were calculated by the NFHS based on sampling probabilities separately for each sampling stage and for each cluster.

The sampling weight for each household in cluster *i* of stratum *h* is the inverse of its overall selection probability: W_*hi*_ = 1/P_*hi*_.

Where, P_*hi*_ is the second stage sampling probability withing the i^th^ cluster.

The household sampling weight was further adjusted by the NFHS for individual non-response to obtain individual sampling weight separately for men and women. These sampling weights were normalized at the national level to obtain national standard weights and at the state level to obtain state standard weights. We multiplied the state standard weights with tobacco percentage to obtain age-specific tobacco prevalence for men and women.

To account for differences in the population structure across states and time, we age-standardized the sex-specific tobacco prevalence using direct standardization, using the Indian Census population of 2011 as the standard population. We mapped differences in age-standardized tobacco prevalence across states and by sex. We classified our data according to five equally large intervals. We tested whether the differences in the age-standardized sex specific prevalence between the states and in the country as a whole are significant [36]. We assessed the significance level of the difference in the age-standardized rates between states and India as a whole using the formula for the Z-score of the difference between proportions, assuming a normal distribution.

All analyses were performed using Stata15.

## Results

**Fig. 1:**
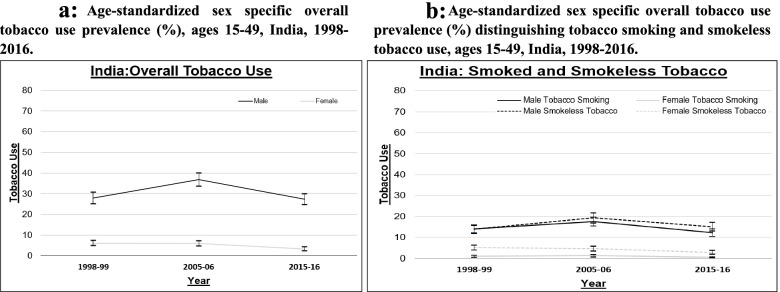
Age standardized sex specific overall tobacco use prevalence in India, ages 15–49 years, 1998-99. Legend: Data Source: NFHS India, (Round II -IV). Own calculation

The age-standardized tobacco use prevalence among men in India increased by 32% between 1998 and 99 and 2005-06 (from 27.90 to 36.81%), and declined by 26% between 2005 and 06 and 2015–2016 (from 36.81 to 27.31%), whereas the age-standardized prevalence of tobacco use among women continued to decline from 1998 to 99 to 2015-16 (Fig. 1a). Between 1998 and 99 and 2005-06, the age-standardized smoking prevalence increased by 25% for men (from 14.07 to 17.54%) and by 32% for women (from 0.93 to 1.24%), whereas the age-standardized prevalence of smokeless tobacco use also increased for men (by 39% from 13.83 to 19.28%), but declined slightly for women (by 9% from 5.06 to 4.62%). Between 2005 and 06 and 2015-16, the age-standardized smoking prevalence declined to 12.20% for men and to 0.40% for women, and the age-standardized prevalence of smokeless use tobacco declined to 15.12% for men and 2.87% for women (Fig. 1b). Despite these declines, the prevalence of tobacco use remains high; at a level comparable to that observed in 1998-99 for both men and women. While men have a higher prevalence of both smokeless tobacco and tobacco smoking; use of smokeless tobacco is more extensive than tobacco smoking among women.

**Fig. 2:**
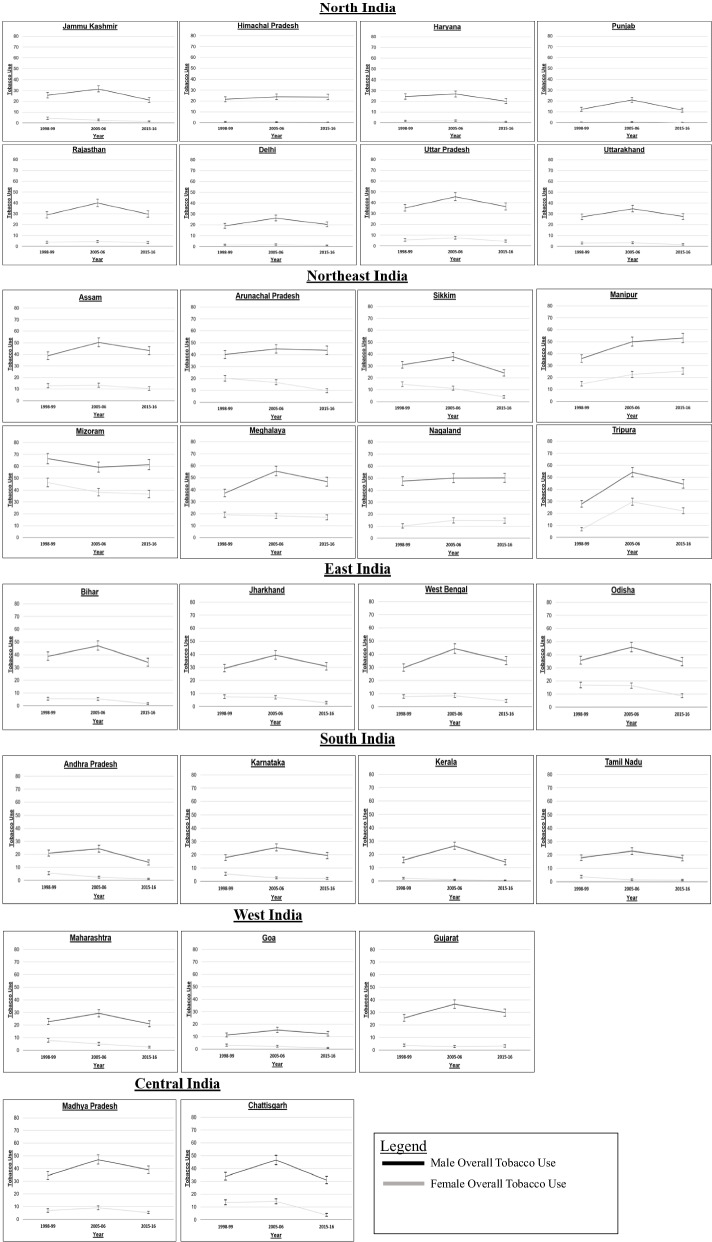
Trends in the prevalence of age-standardized sex-specific tobacco use (%), ages 15–49, by sex, India, 1998–2016. Legend: Data Source: NFHS India, (Round II-IV). Own calculation. Note: The scales for women and men are different, but are consistent for all states in order to compare levels across states. The progression of the tobacco epidemic could not be provided for one state (Telangana) because the state was newly formed in 2014, or for six union territories (Andaman and Nicobar Islands, Chandigarh Daman and Diu and Dadra and Nagar Haveli, Lakshadweep, Ladakh and Puducherry)

There are marked differences in tobacco use patterns at the regional level compared to the national level. Exceptions to the overall declining trends after 2005 can be observed for men in the north-eastern regions (states of Manipur, Mizoram, and Nagaland) and women in the north-eastern and the western regions (states of Gujarat and Manipur), where the prevalence of tobacco use still increases (Fig. 2). Among women in Gujarat, this increasing trend has been discernible even though the level of tobacco use in the state has been very low.

Similarly, important regional differences in trends were detected when we dichotomize tobacco use into tobacco smoking and smokeless tobacco (Fig. S1). Tobacco smoking is increasing among men in the northern and north-eastern regions (states of Himachal Pradesh, Arunachal Pradesh, Manipur, and Meghalaya) and among women in the southern regions (states of Karnataka and Tamil Nadu), whereas smokeless tobacco use is increasing among men in the north-eastern regions (states of Manipur, Mizoram, and Nagaland) and among women in the northern, north-eastern, and western regions (states of Rajasthan, Manipur, and Gujarat).

**Fig. 3:**
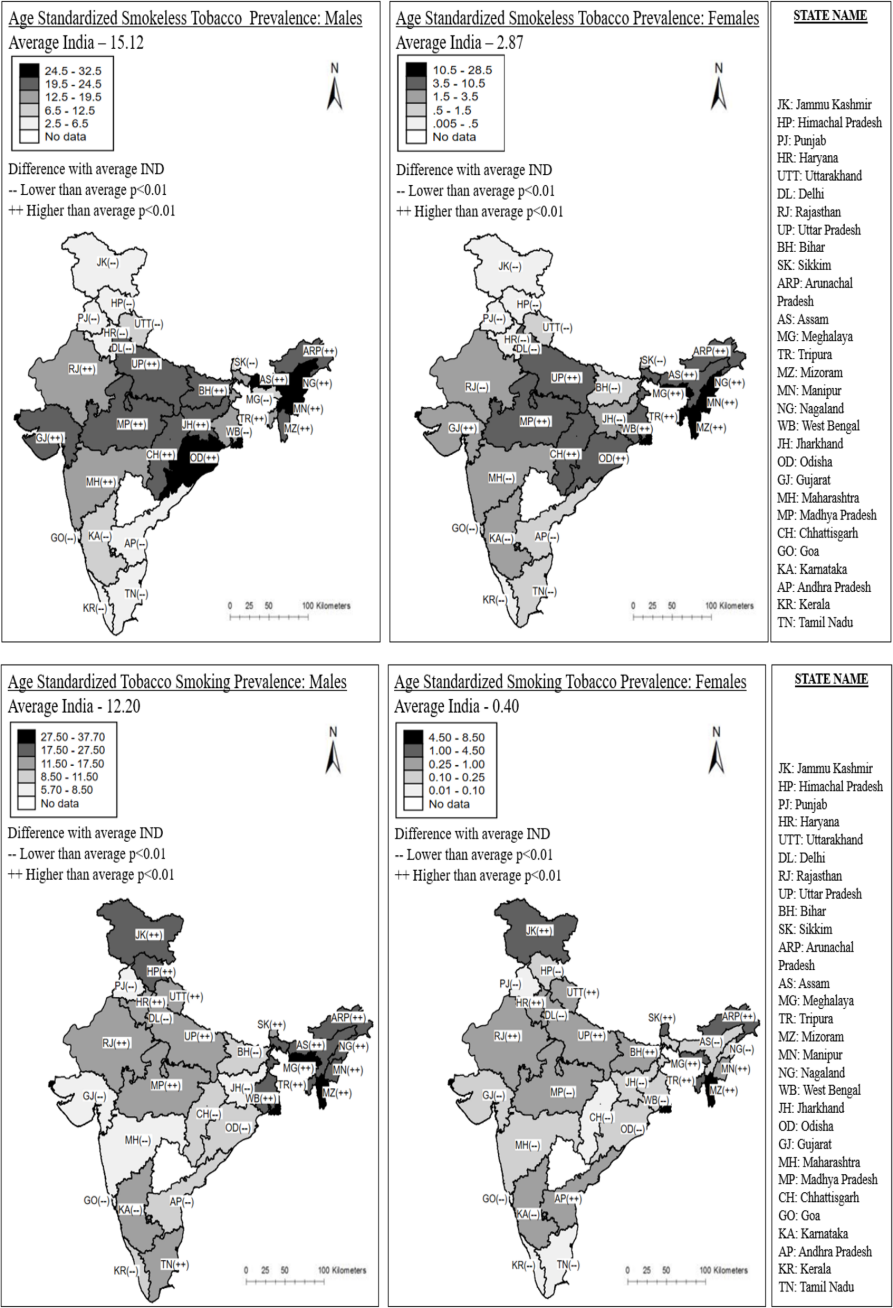
Regional differences in the prevalence of age-standardized sex-specific tobacco use (%), distinguishing between tobacco smoking and smokeless tobacco use, ages 15–49, by sex, India, 1998–2016. Legend: Data Source: NFHS India, (Round II-IV). Own calculation

While in most of the Indian states, levels of tobacco consumption have recently been declining, we can observe clear regional differences in the current patterns of smoking and smokeless tobacco use (Fig. 3). Among men and women in north-eastern states, both tobacco smoking and smokeless tobacco use are more prevalent than the national average. We also find that in the southern Indian states, prevalence of tobacco consumption is significantly lower than the national average. In 2015-16, male smoking prevalence ranged from 5.71% in Maharashtra in the west to 37.70% in Mizoram in the north-east, while female smoking prevalence ranged from 0.01% in Kerala in the south to 8.45% in Mizoram in the north-east. Similarly, the prevalence of smokeless tobacco use among men ranged from 2.64% in Kerala in the south to 32.01% in Nagaland in the north-east, while the prevalence of smokeless tobacco use among women ranged from 0.02% in Himachal Pradesh in the north to 28.18% in Mizoram in the north-east.

## Discussion

### Summary of results

Our analysis revealed that in India, the age-standardized prevalence of tobacco use at national level has been higher among men than women: i.e., it increased between 1998-1999 and 2005-2006 and declined from 2005-2006 to 2015–2016 among men, whereas it declined continuously among women. While for most of the states we observed a declining trend in tobacco use after 2005, men in the north-eastern states of Manipur, Mizoram, and Nagaland and women in the western state of Gujarat and the north-eastern state of Manipur showed an increasing trend in tobacco use. Similarly, we observed clear regional differences in tobacco smoking and smokeless tobacco over time, with more states showing an increasing prevalence in either tobacco smoking or smokeless tobacco. Prevalence of tobacco use has been higher than the national average for the north-eastern states as compared to other states/regions of India, and has been higher among men than women for all regions.

### Discussion of results

Our findings that the prevalence of tobacco use first increased and then declined over time, and that the prevalence of smoking was higher among men than women, are in line with the classical smoking epidemic theory [5]. According to the classical smoking epidemic model, the increase and later decline in smoking prevalence took more than 5–6 decades. occurring first among men and only 2–3 decades later among women, and with a maximum smoking prevalence of approximately 55% among men and approximately 32% among women. Such an extended smoking epidemic pattern with clear differences in timing between men and women has been observed for the majority of European and North American/Australasian countries [37], and in developing countries like China [6].

India, on the other hand, has a pattern that is quite distinct from the classical smoking epidemic model. The maximum levels of smoking prevalence for both men and women in India (17.5% for men; 1.2% for women) are considerably lower than those anticipated in the classical smoking epidemic model. In addition, the approximately similar timing of the increase and decrease in tobacco smoking among men and women differs from the patterns found in western countries. Moreover, the rather low prevalence levels in 1998/99 seem to suggest that India underwent an increase followed by a decline in the prevalence of tobacco use over a comparatively short period of time. In light of these apparent deviations from the classical smoking epidemic model, we suggest that India has experienced a ‘compressed tobacco epidemic’.

A potential explanation for the compression of the tobacco epidemic in India is that larger numbers of Indians did not take up smoking until the late 20th century, at a time when the hazards of tobacco consumption were well understood. By contrast, men in Europe and North America/Australasia first took up smoking in the first half of the 20th century, when the negative health consequences were largely unknown [5, 37]. Thus, tobacco use was increasing among Indians at a time when the negative effects of tobacco consumption on health were already very apparent [7], and prevention strategies had been established [38–40]. The lack of social acceptance of tobacco use among women and increase in the price of smokeless tobacco [41] are among other factors that may have contributed to the prevalence of both, tobacco smoking and smokeless tobacco use being substantially lower among women in India than their counterparts in other countries [42, 43]. In addition, India stands out as one of the countries where prevalence of tobacco consumption has been substantially reduced since 2005 [44] through the introduction of a large number of tobacco control policies (Figure S2). For example, the country implemented the National Tobacco Control Programme (NTCP) in 2008 with the aim of reducing the burden of tobacco dependence [45]. More recently, India initiated an mCessation service, a mobile service designed to motivate people to quit smoking [46]. The implementation of these policies on a nationwide scale has helped to reduce tobacco consumption, which has, in turn, contributed to the compression of the tobacco epidemic.

We also expanded the classical smoking epidemic model by including the use of smokeless tobacco as an important, country-specific form of tobacco consumption. On a national level in India, smokeless tobacco consumption follows the trends for tobacco smoking, and therefore fits into the classical smoking epidemic framework. Although the maximum levels of smokeless tobacco use prevalence (19.28% for men and 5.06% for women) have been substantially lower in India than in other countries [46], they were higher than the prevalence of tobacco smoking for all of the time points, particularly for women. Thus, the use of smokeless tobacco cannot be ignored when studying the tobacco epidemic in India.

While for most of the states we observed a declining trend in tobacco use after 2005, men and women in few states showed an increasing trend in tobacco use. Although overall tobacco use increased only for 4 Indian states (Manipur, Mizoram, Nagaland and Gujarat); tobacco smoking and/or smokeless tobacco increased in two north, five north-eastern, 2 southern and one western Indian state. Increase in overall tobacco use in states can be attributed to increase in tobacco smoking among men in Manipur (10.85% increase) and increase in smokeless tobacco use among men in Mizoram (15.81% increase) and Nagaland (10.5% increase), and women in Gujarat (27.89% increase) and Manipur (22.71% increase) (Table S2). The continued rise in tobacco use in these states are most likely be related to social and environmental influence such as parental influence, lower educational status, attraction towards role models and cultural practices. That is, the continued rise in tobacco use in the north-eastern region is likely to a strong degree attributed to the high social acceptance of tobacco use and cultural practices [24, 31], less effective state policies [32] and greater availability of tobacco products [31, 32]. The continued increase in tobacco use among women in Gujarat is most likely due the fact that this state is the second largest producer of tobacco [47], and has a high employment of women in this sector [48].

A further explanation for the relatively high tobacco consumption levels currently observed in India is related to the tobacco regulations that were implemented in the 1990 and 2000 s, which were less effective in targeting bidi smoking and smokeless tobacco use [31, 32]. Indeed, additional analysis revealed that smoking prevalence is still relatively high in states with bidi smoking traditions (Table S1). In these states, women tend to smoke more bidis and use more smokeless tobacco, while smoking fewer cigarettes (Table S1). This pattern may be partially explained by the comparatively high levels of employment of women in the bidi industry [48], and by women’s preferences due their economic constraints to consume more bidis and smokeless tobacco than cigarettes [42]. Prevalence of tobacco use was observed to be highest among men and women in the north-eastern states as compared to other states in the country. In these states, prevalence of tobacco smoking and smokeless tobacco use was increasing continuously among men and was declining slowly among women, resulting in tobacco use prevalence that was much higher than the national average. These large regional difference may be a result of state specific higher cultural and societal acceptance of tobacco use [24, 31]; greater access and availability of tobacco products [31, 32]; less effective implementation of state policies, and lower levels of adherence to these policies [32]; and increased female employment in the tobacco sector [49]. Different geographical and particularly socio-economic differences between and within states may also be a driver for increased tobacco use in states [24, 31, 32]. Individuals with lower education status and belonging to less favourable economic strata are more likely to use tobacco [24]. Additionally, socioeconomic differences were seen to be more prominent for tobacco smoking than smokeless tobacco [24].

Despite the overall progress India made in reducing tobacco consumption, it remains a public health priority, not only because of the situation in the north-eastern regions, but also because in India as a whole, the levels of tobacco use in 2015–2016 were largely comparable to the levels in 1998–1999 (Table S2), and smokeless tobacco and bidi consumption still play a major role in many parts of the country (Table S1). The complex tax structure in India has kept taxes on cigarettes relatively low, and taxes on bidis and smokeless tobacco products very low, compared to other countries [41, 50]. Thus, bidis and smokeless tobacco products remain quite affordable. Whereas taxes on cigarettes account for approximately 38% of the retail price, taxes on bidis account only 9% of the retail price. These taxes are well below the tax rates on tobacco products recommended by the World Bank of 65–80% of the retail price [50, 51]. In 2011 in India, the price of a single cigarette was US$0.026, whereas the price of a single bidi was US$0.0033^51,52^. Similarly, in 2016, retail price per pack of most brands of smokeless tobacco was US$0.10^37^. A study by John et al. (2007) suggested that approximately 23 million people would stop smoking bidis and 4.7 million people would stop smoking cigarettes if taxes were increased from US$0.19 to US$1.33 per 1000 bidis (9–40% of the retail price) and from US$8.98 to US$50.27 per 1000 cigarettes (from 38 to 78% of the retail price) [51, 52].

### Evaluation of data and methods

Our study is the first to provide insights into the progression of the tobacco epidemic by gender across Indian states. For our study, we used several rounds of the NFHS to estimate the age-standardized smoking and smokeless tobacco prevalence. Specifically, we used information on tobacco consumption for men and women between 15 and 49 years of age, as the cumulative tobacco consumption levels between these ages are a strong predictor of adverse health consequences later in life [53, 54]. The NFHS has a huge sample size and uses the same sampling procedure across its different rounds to provide relatively accurate representation of the population at national and state levels [33], which is not the case in other sample surveys, like the Global Adult Tobacco Survey (GATS), the Global Youth Tobacco Survey (GYTS), and the National Household Survey of Drug and Alcohol Abuse in India (NHSDAA). For example, the GATS, which was used in the studies by Mishra et al. (2016) and Goel et al. (2014), has considerable under-sampling in the 10 states with highest levels of tobacco consumption [55]. In addition, discrepancies in reporting of the procedures used for data collection and quality assurance in GATS may have significantly affected its estimates of tobacco use prevalence [56]. Similarly, the NHSDAA covered only men from 25 states [57], and the GYTS was designed to collect information on tobacco use among young people between ages 13–15 only [58]. Moreover, the NHSDAA was conducted at only one point in time, while the GYTS was conducted at just two points in time. Using the NFHS for the present study was advantageous, as it is a repeated cross-sectional survey representing 99% of the Indian population. Prevalence of tobacco consumption was captured in the NFHS rounds using self-administered questionnaires, which meets the global standardized guidelines [59].

However, some limitations of our study should be taken into account when interpreting our results. First, we provide information only on current tobacco use, and not on the severity (duration/amount) of the consumption. This latter information is, unfortunately, not provided by the NFHS. Still, we believe that for studying the tobacco epidemic, the information provided by NFHS is reliable. Second, because the NFHS survey rounds were conducted with substantial time lags, the exact timing of the peak of the tobacco epidemic cannot be assessed using this data, especially among females. Third, the overall prevalence of tobacco use in the Indian population may be higher [44], as we focus only on 15–49 age group. Finally, the present study considered only the prevalence of tobacco use, while the classical smoking epidemic model also includes smoking-attributable mortality. This is certainly an important field for future research that will provide further insights into the overall health burden of tobacco smoking and smokeless tobacco in India. Another important perspective is to look into socio-economic differences within and across states and how they contribute to regional and national tobacco consumption patterns.

## Conclusions

Our results suggest that India and the majority of its states have experienced a ‘compressed tobacco epidemic’ which is quite distinct from the experiences of western countries. Despite the overall progress India made in reducing tobacco consumption, lowering it further remains a public health priority, as the prevalence of tobacco use in 2015-2016 was highly comparable to the prevalence of 1998-1999, and remains high especially in the north-eastern regions. Consumption of smokeless tobacco and bidis still plays a major role in many parts of the country, which can be linked to the very low taxes on bidis and smokeless tobacco products. We therefore recommend that the taxes and/or the prices be raised for these products in particular, but also for cigarettes. Strict implementation of policies, especially in the north-eastern part of the country, would substantially reduce the overall prevalence of tobacco use in India, and, in turn, the burden of tobacco-related morbidity and associated premature mortality in the country.

## Supplementary Information

**Additional file 1.**

## Data Availability

The datasets generated and/or analysed during the current study are available from the Demographic and Health Survey (DHS) website (Link: The DHS Program - Available Datasets).

## Abbreviations

NFHS: National Family Health Survey
WHO: World Health Organization
NTCP: National Tobacco Control Programme
GATS: Global Adult Tobacco Survey
GYTS: Global Youth Tobacco Survey
NHSDAA: National Household Survey of Drug and Alcohol Abuse
