# Supplementary material for: The progression of the tobacco epidemic in India on the national and regional level, 1998-2016
Source: BMC Public Health. 2022 Feb 15;22:317. doi: 10.1186/s12889-021-12261-y (PMC8845293; doi:10.1186/s12889-021-12261-y)
Supplement: Supplementary file 1 — Additional file 1. [file 12889_2021_12261_MOESM1_ESM.docx]

Supplementary File

The progression of the tobacco epidemic in India on the national and regional levels, 1998-2016

**Corresponding Author:** Dr.Tobias Vogt

## Supplementary Figures and Tables

**Figure S1:** Trends in the prevalence of age- and sex-standardized tobacco use (%), distinguishing between tobacco smoking and smokeless tobacco use, ages 15-49, by sex, India, 1998-2016.

**Figure S2:** Roadmap to Smoking Policies in India.

**Table S1:** Age-and-sex standardized prevalence of cigarette and bidi smoking and smokeless tobacco consumption, by sex , between ages 15-49 years, Indian states, 2015-16.

**Table S2:** Percent decline in age-and-sex standardized tobacco prevalence (2005-06 to 2015-16), by sex, between ages 15-49 years of age, India.

**Figure S1:** Trends in the prevalence of age- and sex-standardized tobacco use (%), distinguishing between tobacco smoking and smokeless tobacco use, ages 15-49, by sex, India, 1998-2016.


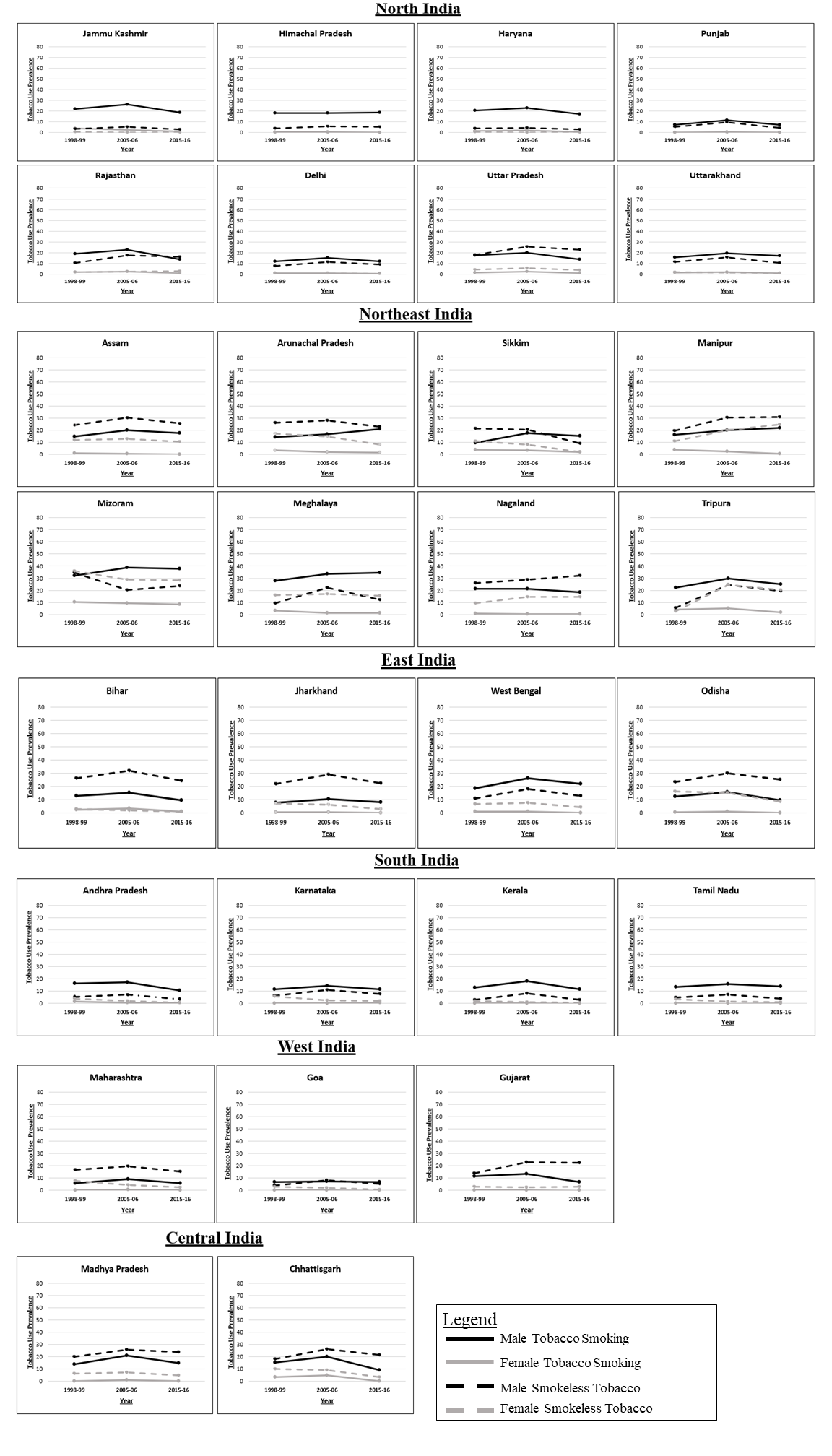


Legend: Data Source: NFHS India, (Round II-IV). Own calculation

Note: The scales for women and men are different, but are consistent for all states in order to compare levels across states.

The progression of the tobacco epidemic could not be provided for one state (Telangana) because the state was newly formed in 2014, or for six union territories (Andaman and Nicobar Islands, Chandigarh Daman and Diu and Dadra and Nagar Haveli, Lakshadweep, Ladakh and Puducherry).

**Figure S2:** Roadmap to Smoking Policies in India


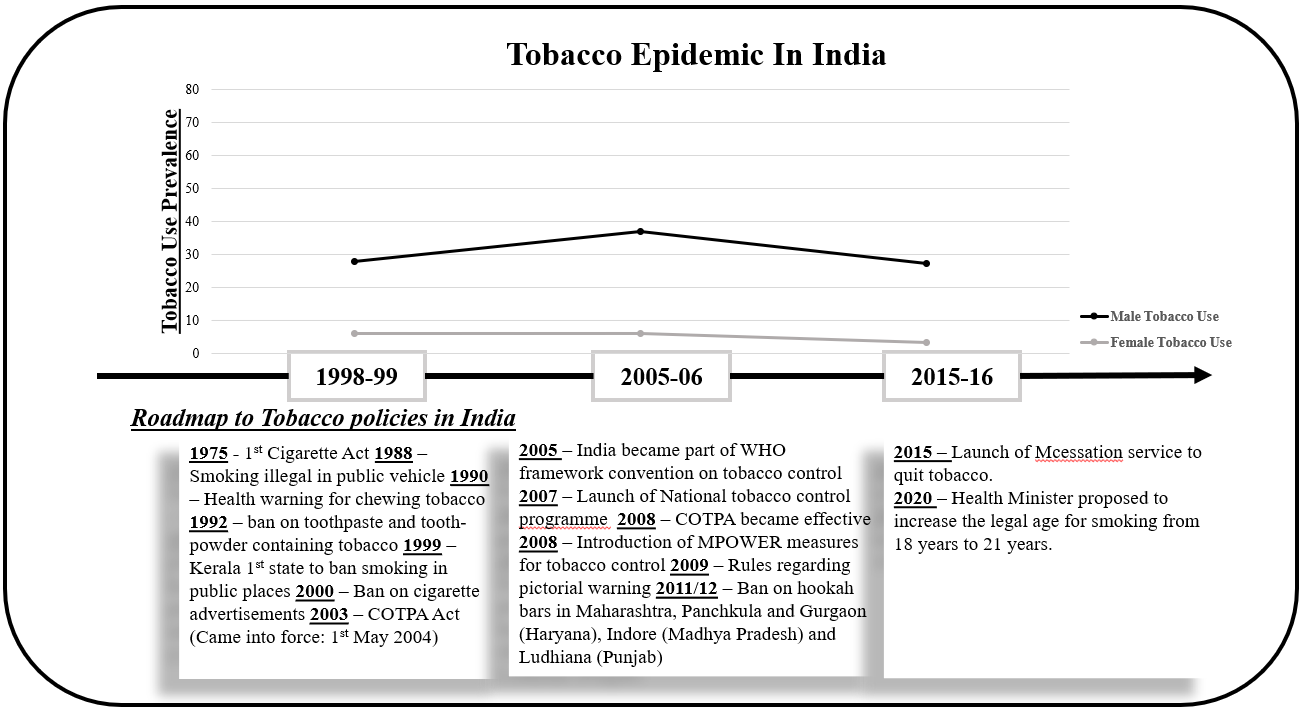


Legend: Data Source: NFHS (Round II-IV)

Sources used to make the roadmap: Tobacco Control in India, WHO, and National Tobacco Control Programme.

**Table S1**: Age-and-sex standardized prevalence of cigarette and bidi smoking and smokeless tobacco consumption, by sex, between ages 15-49 years, Indian states, 2015-16.

| **Region** | **Indian State** | **Men 2015-16*** | | | **Women 2015-16*** | | | |
| --- | --- | --- | --- | --- | --- | --- | --- | --- |
|  |  | **Cigarette Smoking** | **Bidi Smoking** | **Smokeless Tobacco** | **Cigarette Smoking** | **Bidi Smoking** | **Smokeless Tobacco** | |
| North | Jammu and Kashmir | 15.3(17.35,13.25) | 4.12(5.15,3.08) | 2.83(3.7,1.96) | 0.15(0.34,-0.04) | 0.15(0.34,-0.04) | | 0.25(0.49,0) |
|  | Himachal Pradesh | 12.53(14.40,10.66) | 14.23(16.17,12.29) | 5.22(6.42,4.01) | 0.05(0.17,-0.07) | 0.2(0.41,-0.02) | | 0.02(0.08,-0.05) |
|  | Punjab | 3.77(4.81,2.73) | 4.55(5.64,3.45) | 4.26(5.32,3.2) | 0.01(0.06,-0.04) | 0.04(0.15,-0.06) | | 0.02(0.1,-0.05) |
|  | Haryana | 4.62(5.79,3.45) | 13.26(15.11,11.41) | 2.94(3.87,2.02) | 0.08(0.21,-0.06) | 0.55(0.9,0.19) | | 0.19(0.41,-0.03) |
|  | Rajasthan | 3.78(4.83,2.73) | 10.58(12.21,8.95) | 16.19(18.33,14.04) | 0.02(0.09,-0.05) | 0.75(1.17,0.33) | | 2.48(3.31,1.65) |
|  | Uttar Pradesh | 5.63(6.91,4.36) | 9.9(11.48,8.32) | 22.63(25.13,20.13) | 0.03(0.11,-0.06) | 0.58(0.95,0.21) | | 3.53(4.48,2.58) |
|  | Uttarakhand | 7.69(9.17,6.21) | 11.4(13.09,9.71) | 10.37(12.08,8.67) | 0.06(0.17,-0.06) | 0.76(1.18,0.34) | | 0.84(1.30,0.39) |
|  | Delhi | 6.16(7.51,4.82) | 6.46(7.75,5.16) | 8.67(10.21,7.13) | 0.14(0.34,-0.05) | 0.24(0.47,0.01) | | 0.57(0.94,0.19) |
| North-East | Assam | 11.19(13,9.39) | 8.61(10.14,7.08) | 25.59(28.28,22.91) | 0.02(0.11,-0.06) | 0.06(0.18,-0.06) | | 10.27(11.92,8.61) |
|  | Arunachal Pradesh | 17.41(19.66,15.16) | 9.51(11.14,7.87) | 22.85(25.39,20.31) | 0.42(0.77,0.07) | 0.64(1.04,0.24) | | 8.22(9.71,6.73) |
|  | Sikkim | 14.59(16.68,12.50) | 1.1(1.66,0.54) | 8.87(10.43,7.31) | 1.57(2.26,0.88) | 0.58(0.94,0.21) | | 2(2.71,1.30) |
|  | Manipur | 20.63(23.05,18.21) | 3.83(4.85,2.82) | 31.02(33.98,28.07) | 0.32(0.59,0.05) | 0.39(0.69,0.08) | | 24.59(27.19,21.99) |
|  | Mizoram | 35.68(38.87,32.48) | 5.33(6.56,4.10) | 23.64(26.22,21.06) | 7.36(8.77,5.94) | 0.76(1.21,0.31) | | 28.18(31,25.36) |
|  | Meghalaya | 24.17(26.78,21.55) | 24.76(27.42,22.11) | 12.12(13.97,10.28) | 0.25(0.52,-0.02) | 0.75(1.71,0.34) | | 15.66(17.74,13.59) |
|  | Nagaland | 10.99(12.79,9.19) | 12.41(14.27,10.54) | 32.01(35.06,28.96) | 0.1(0.28,-0.08) | 0.04(0.13,-0.06) | | 14.34(16.38,12.30) |
|  | Tripura | 14.8(16.83,12.77) | 14.73(16.69,12.78) | 19.36(21.68,17.03) | 0(0.04,-0.03) | 1.3(1.86,0.74) | | 20.45(22.78,18.11) |
| East | Bihar | 8.04(9.55,6.53) | 2.55(3.34,1.75) | 24.33(26.9,21.76) | 0.09(0.24,-0.06) | 0.68(1.08,0.29) | | 0.78(1.22,0.33) |
|  | Jharkhand | 6.62(8.01,5.24) | 2.65(3.49,1.81) | 22.59(25.07,20.11) | 0.02(0.08,-0.05) | 0.05(0.16,-0.06) | | 2.7(3.51,1.88) |
|  | Odisha | 6.47(7.82,5.13) | 4.64(5.73,3.55) | 25.2(27.83,22.56) | 0.02(0.09,-0.05) | 0.06(0.17,-0.06) | | 8.36(9.81,6.91) |
|  | West Bengal | 12.68(14.59,10.78) | 16.6(18.73,14.47) | 13.08(14.97,11.18) | 0.04(0.14,-0.07) | 0.15(0.33,-0.04) | | 4.12(5.14,3.09) |
| South | Andhra Pradesh | 7.91(9.37,6.44) | 3.51(4.44,2.58) | 3.18(4.1,2.27) | 0.01(0.08,-0.05) | 0.06(0.19,-0.06) | | 0.58(0.96,0.2) |
|  | Karnataka | 9.05(10.65,7.46) | 4.01(5,3.02) | 7.73(9.17,6.28) | 0.18(0.40,-0.05) | 0.04(0.14,-0.07) | | 1.72(2.37,1.06) |
|  | Kerala | 10.92(12.66,9.19) | 2.49(3.29,1.70) | 2.64(3.51,1.78) | 0.01(0.06,-0.04) | 0(0,0) | | 0.34(0.63,0.04) |
|  | Tamil Nadu | 8.69(10.24,7.14) | 6.38(7.66,5.10) | 3.8(4.83,2.77) | 0.02(0.11,-0.06) | 0.02(0.09,-0.05) | | 0.91(1.37,0.45) |
| West | Maharashtra | 4.75(5.91,3.60) | 1.17(1.70,0.63) | 15.23(17.27,13.19) | 0.03(0.13,-0.07) | 0.01(0.07,-0.04) | | 2.36(3.14,1.58) |
|  | Goa | 6.12(7.45,4.79) | 0.91(1.39,0.43) | 5.35(6.55,4.15) | 0.04(0.17,-0.08) | 0(0,0) | | 0.76(1.20,0.33) |
|  | Gujarat | 2.47(3.31,1.62) | 4.69(5.76,3.63) | 22.81(25.34,20.28) | 0.01(0.06,-0.04) | 0.07(0.19,-0.06) | | 3.21(4.15,2.27) |
| Central | Chhattisgarh | 5.93(7.27,4.59) | 4.9(6.03,3.77) | 21.75(24.22,19.29) | 0.02(0.09,-0.06) | 0.04(0.15,-0.06) | | 3.74(4.72,2.76) |
|  | Madhya Pradesh | 6.22(7.57,4.86) | 10.93(12.61,9.24) | 23.78(25.93,21.63) | 0.02(0.10,-0.06) | 0.13(0.31,-0.04) | | 5.01(5.84,4.19) |
| **India** | | 7.15(8.57,5.73) | 6.76(8.08,5.44) | 15.12(17.17,13.07) | 0.05(0.16,-0.07) | 0.23(0.46,0) | 2.87(3.73,2.01) | |

Legend: *Values in the bracket represent 95% CI. Yellow indicates states with more bidi consumption than cigarette.

Data Source: NFHS (2015-16). Own calculation

**Table S2**: Percent Decline in age-and-sex standardized tobacco prevalence (2005-06 to 2015-16), by sex, between ages 15-49 years, India.

|  |  | **Men Tobacco Use (95%CI)** | | **Women Tobacco Use (95%CI)** | |
| --- | --- | --- | --- | --- | --- |
| **Region** | **Indian State** | **Smoking** | **Smokeless** | **Smoking** | **Smokeless** |
| North | Jammu and Kashmir | 29.52(33,26.04) | 48.16(49.68,46.63) | 51.32(52.28,50.37) | 1.32(1.67,0.97) |
|  | Himachal Pradesh | -2.11(1.01,-5.23) | 9.35(11.10,7.59) | 63.64(64.09,63.19) | 23.38(23.47,23.29) |
|  | Punjab | 36.62(38.89,34.35) | 55.42(57.38,53.46) | 83.71(84.01,83.40) | 77.34(77.52,77.16) |
|  | Haryana | 24.42(27.67,21.17) | 27.79(29.22,26.36) | 60.09(60.84,59.34) | 12.03(12.35,11.71) |
|  | Rajasthan | 40.29(43.39,37.18) | 6.58(9.69,3.46) | 55.83(56.71,54.96) | -13.79(-12.65,-14.92) |
|  | Uttar Pradesh | 30.62(33.62,27.62) | 11.88(15.56,8.20) | 60.23(61.08,59.39) | 35.38(36.9,33.85) |
|  | Uttarakhand | 10.32(13.42,7.21) | 33.87(36.58,31.16) | 51.76(52.54,50.98) | 40.11(40.86,39.36) |
|  | Delhi | 21.45(24.13,18.78) | 23.85(26.23,21.47) | 59.39(59.93,58.84) | 33.40(34.01,32.79) |
| North-East | Assam | 11.32(14.58,8.05) | 16.33(20.31,12.34) | 78.36(78.74,77.97) | 19.86(22.33,17.39) |
|  | Arunachal Pradesh | -23.16(-19.85,-26.47) | 18.16(21.94,14.37) | 30.62(31.58,29.67) | 44.28(46.77,41.79) |
|  | Sikkim | 13.15(16.26,10.05) | 56.56(59.39,53.74) | 36.7(37.88,35.52) | 75.10(76.72,73.48) |
|  | Manipur | -10.85(-7.43,-14.28) | -2.62(1.49,-6.83) | 72.18(73.04,71.32) | -22.71(-19.19,-26.23) |
|  | Mizoram | 2.83(7.52,-1.87) | -15.81(-12.3,-19.33) | 8.44(10.62,6.26) | 2.72(6.74,-1.31) |
|  | Meghalaya | -2.86(1.52,-7.24) | 44.91(48.04,41.79) | 9.58(10.37,8.8) | 6.93(9.93,3.94) |
|  | Nagaland | 13.78(17.12,10.45) | -10.5,(-6.3,-14.7) | 51.57(51.95,51.18) | 0.18(3.08,-2.71) |
|  | Tripura | 15.47(19.33,11.61) | 21.26(24.8,17.73) | 65.13(66.42,63.83) | 17.05(20.54,13.56) |
| East | Bihar | 36.7(39.34,34.07) | 23.33(27.26,19.4) | 74.6(75.61,73.59) | 58.82(59.65,57.99) |
|  | Jharkhand | 20.75(23.05,18.45) | 22.26(26.03,18.5) | 82.35(82.79,81.91) | 57.07(58.6,55.55) |
|  | Odisha | 39.84(42.45,37.23) | 15.88(19.8,11.95) | 84.22(84.78,83.65) | 45.16(47.64,42.68) |
|  | West Bengal | 15.76(19.41,12.12) | 28.12(31.1,25.14) | 76.09(76.59,75.59) | 46.15(47.91,44.39) |
| South | Andhra Pradesh | 38.37(41.07,35.66) | 55.44(57.15,53.74) | 2.98(3.45,2.5) | 68.71(69.48,67.93) |
|  | Karnataka | 19.62(22.27,16.97) | 29.19(31.47,26.91) | -312.18(-311.81,-312.54) | 30.53(31.55,29.5) |
|  | Kerala | 37.08(39.93,34.23) | 67.54(69.31,65.77) | 65.43(65.54,65.31) | 55.23(55.76,54.7) |
|  | Tamil Nadu | 11.7(14.52,8.88) | 47.27(49.03,45.52) | -64.77(-64.6,-64.94) | 28.91(29.64,28.18) |
| West | Maharashtra | 39.74(41.76,37.71) | 23.11(26.22,20) | 76.42(76.92,75.91) | 44.85(46.16,43.54) |
|  | Goa | 4.43(6.39,2.46) | 34.05(35.98,32.12) | 77.27(77.59,76.94) | 61.14(61.97,60.31) |
|  | Gujarat | 47.61(49.91,45.31) | 1.81(5.44,-1.82) | 67.32(67.66,66.99) | -27.89(-26.63,-29.15) |
| Central | Chhattisgarh | 54.09(56.94,51.23) | 17.71(21.42,14) | 98.18(99.36,97.01) | 60.36(62.24,58.47) |
|  | Madhya Pradesh | 28.18(31.32,25.05) | 8.17(10.38,5.95) | 76.42(77.1,75.74) | 33.85(34.94,32.75) |
| India | | 30.45(33.29,27.62) | 21.58(24.69,18.46) | 67.36(68.01,66.72) | 37.95(39.36,36.55) |

Legend: *Highlighted values indicates states showing increase in smoking prevalence. Values in the bracket represent 95% Confidence Interval.

Data Source: NFHS (2005-06 and 2015-16), Own calculation
